# The In Vitro–In Vivo Safety Confirmation of PEG-40 Hydrogenated Castor Oil as a Surfactant for Oral Nanoemulsion Formulation

**DOI:** 10.3390/scipharm85020018

**Published:** 2017-03-31

**Authors:** Heni Rachmawati, Miranti Anggraeni Novel, Sri Ayu, Guntur Berlian, Olivia Mayasari Tandrasasmita, Raymond Rubianto Tjandrawinata, Kusnandar Anggadiredja

**Affiliations:** 1 School of Pharmacy, Institut Teknologi Bandung, Jalan Ganesha 10, 40132 Bandung, Indonesia; anggraeninovel@gmail.com (M.A.N.); sriayu2097@gmail.com (S.A.); kusnandar@fa.itb.ac.id (K.A.); 2 Research Center for Nanosciences and Nanotechnology, Institut Teknologi Bandung, Jalan Ganesha 10, 40132 Bandung, Indonesia; 3 Dexa Laboratories of Biomolecular Sciences, Jababeka, Cikarang 17530, Indonesia; guntur.berlian@dexa-medica.com (G.B.); olivia.tandrasasmita@dexa-medica.com (O.M.T.); raytjan@yahoo.com (R.R.T.)

**Keywords:** PEG-40 hydrogenated castor oil, self-nanoemulsion, glyceryl monooleate, oral delivery, cytotoxicity

## Abstract

Evaluation on the safety use of high concentration of polyoxyl 40 (PEG-40) hydrogenated castor oil as a surfactant for oral nanoemulsion was performed in Webster mice. As previously reported, nearly 20% of PEG-40 hydrogenated castor oil was used to emulsify the glyceryl monooleate (GMO) as an oil to the aqueous phase. Thermodynamically stable and spontaneous nanoemulsion was formed by the presence of co-surfactant polyethylene glycol 400 (PEG-400). Standard parameters were analyzed for nanoemulsion including particle size and particle size distribution, the surface charge of nanoemulsion, and morphology. To ensure the safety of this nanoemulsion, several cell lines were used for cytotoxicity study. In addition, 5000 mg/kg body weight (BW) of the blank nanoemulsion was given orally to Webster mice once a day for 14 days. Several parameters such as gross anatomy, body weight, and main organs histopathology were observed. In particular, by considering the in vivo data, it is suggested that nanoemulsion composed with a high amount of PEG-40 hydrogenated castor oil is acceptable for oral delivery of active compounds.

## 1. Introduction

Nanoemulsions consist of fine oil-in-water dispersions, having droplets covering the size range of 100–600 nm and thermodynamically stable. Stabilization is mostly due to the presence of a combination of surfactant and co-surfactant [1,2,3,4,5]. Nanoemulsion is successfully formed when a high concentration of surfactant is applied. In the formation of nanoemulsion, the free energy of formation is very low and positive or even negative, which results in thermodynamic spontaneous emulsification [6]. Self-emulsification happens due to the penetration of the aqueous phase into the liquid crystalline (LC) phase that is formed at the oil/surfactant–water interface into which water can penetrate, facilitated by gentle agitation during self-emulsification [1,3,4]. After water penetrates to a certain extent, there is a disruption of the interface and a droplet formation. This LC phase is considered to be responsible for the high stability of the resulted nanoemulsion against coalescence [7,8]. One consideration of developing nanoemulsion as potential drug carriers is applying a high concentration of surfactant. Non-ionic surfactants used for carrier formation and stabilizer include polyoxyl 35 castor oil (Cremophor EL), polysorbate 20 (Tween 20), polyoxyl 40 (PEG-40) hydrogenated castor oil, d-α-tocopherol polyethylene glycol 1000 succinate (TPGS), polysorbate 80 (Tween 80), sorbitan monooleate (Span 80), polyoxyl 40 stearate, Solutol HS-15, and various polyglycolyzed glycerides including Labrasol, Gellucire 44/14, Labrafil M-1944CS, and Labrafil M-2125CS [6,9]. Previously, we had established nanoemulsion formulas using both Tween 20 and PEG-40 hydrogenated castor oil as surfactants for different applications of drug loading (curcumin, insulin, bovine serum albumin, recombinant human interferonα-2b) [6,10,11,12].

This report describes a toxicity study of blank nanoemulsion using PEG-40 hydrogenated castor oil as a surfactant we applied in order to load various active compounds for oral administration. The aim was to confirm the safety of using high concentrations (~20%) of PEG-40 hydrogenated castor oil in the formula, especially when used long-term. Polyoxyl 40 hydrogenated castor oil is a non-ionic solubilizer and emulsifying agent that is obtained by reacting hydrogenated castor oil with ethylene oxide [13]. The main constituent of this product is glyceryl polyethylene glycol oxystearate. Together with fatty acid glyceryl polyglyceryl esters, they form the hydrophobic part of the product.

An acute toxicity test of blank nanoemulsion as our universal drug carrier was carried out in mice. Nanoemulsion was given orally at a single dose of 5000 mg/kg body weight (5 males, 5 females). Observation was performed for 14 days after the administration, and the parameters studied include behavioral changes, mortality or differences in gross anatomy of internal organs, organ indices, and histological analysis.

## 2. Materials and Methods

### 2.1. Materials

Glyceryl monooleate (GMO) was purchased from PT. Tritunggal (Jakarta, Indonesia). Polyoxyl 40 hydrogenated castor oil was purchased from BASF (Ludwigshafen, Germany). Polyethylene glycol 400 (PEG-400) was commercially provided by PT. Bratachem (Bandung, Indonesia). Dulbecco’s Modified Eagle Medium (DMEM), Eagle’s Minimum Essential Medium (EMEM), the Roswell Park Memorial Institute (RPMI) 1640 medium, penicillin–streptomycin, bovine serum, fetal bovine serum, and trypsin–ethylenediaminetetraacetic acid (EDTA) were purchased from Gibco (Waltham, MA, USA). 3-(4,5-Dimethylthiazol-2-yl)-5-(3-carboxymethoxyphenyl)-2-(4-sulfophenyl)-2*H*-tetrazolium (MTS) reagents were purchased from Promega (Madison, WI, USA).

### 2.2. Cell Line

NIH/3T3 (mouse fibroblast cell line, CRL-1658), 3T3-SA (Swiss albino mouse fibroblast cell line, CCL-92), RSC-96 (rat neuron Schwann cell line, CRL-2765), RAW 264.7 (mouse macrophage cell line, TIB-71), RBL-2H3 (rat basophilic leukemia cell line, CRL-2256), CHO-K1 (Chinese hamster ovary cell line, CCL-61), NCI-H292 (human tumor cell line, CRL-1848), and Caco-2 (human epithelial colorectal adenocarcinoma cell) were purchased from American Type Culture Collection (ATCC) (Rockville, MD, USA).

### 2.3. Animal

Specific, pathogen-free male and female Webster mice weighing 25–30 g at the beginning of experiment were used. Animal were obtained from School of Pharmacy, Bandung Institute of Technology, Indonesia. The mice received a standard diet and were housed under standard laboratory conditions. The study as presented was approved by the Local Committee for Care and Use of Laboratory Animals, School of Pharmacy, Bandung Institute of Technology, Indonesia (Certificate No. 08/KEPHP-ITB/032015) and was performed according to strict governmental and international guidelines on animal experimentation.

### 2.4. Preparation of Blank Nanoemulsion

Blank nanoemulsion consisted of an oil phase of GMO, PEG-40 hydrogenated castor oil and PEG-400 (1:8:1) was prepared using our established formula [6]. The mixture of oil, surfactant and co-surfactant were stirred at 100 rpm for 2 h. Further sonication for 1 h using a bath sonicator (Nagoya S Ultrasonic Cleaner GB-928, Shenzhen Co.Ltd, Guangdong, China) was applied to complete the mixing process. To obtain nanoemulsion, deionized water was added to the oil phase at a ratio of 5:1 and stirred gently. Blank nanoemulsion was spontaneously formed after this protocol.

### 2.5. Physical Characterization of Blank Nanoemulsion

Characterization of blank nanoemulsion was performed as previously [6]. Briefly, the successful formation of nanoemulsion was evaluated based on analysis of particle size and particle size distribution, and zeta potential using particle size analyzer (Delsa Nano C Particle Analyzer, Beckman Coulter, Brea, CA, USA).

Particle morphology of the nanoemulsion was analyzed by transmission electron microscopy (TEM) using a JEM-1400 transmission electron microscope (JEOL, Tokyo, Japan). About 10 mL of sample was dropped in the specimen place and covered with a 400 mesh grid. After 1 min, 10 mL of uranyl acetate was dropped on top of the grid, and this sample was allowed to dry for 30 min before observation under the transmission electron microscope. This procedure was used to confirm the particle size in the nanoemulsion as measured using the particle size analyzer.

### 2.6. Stability Study of Blank Nanoemulsion in Gastrointestinal Tract Simulation Fluids

The proposed delivery system of this nanoemulsion is for oral route. In order to ensure the physical stability of the nanoemulsion, the influence of gastrointestinal tract (GIT) condition was evaluated using both gastric simulation fluid (0.1 N HCl) and intestinal simulation fluid (phosphate buffer at pH 6.8) at 37 °C for 3 h, respectively. Same volume ratio of nanoemulsion and GIT simulation fluid was mixed and observation was done visually and by measuring the particle size as previously described.

### 2.7. Stability Study of Blank Nanoemulsion during Storage

The physical stability of the blank nanoemulsion was performed by storing the sample at room temperature for nine months. The observation was done visually and by measuring the particle size as previously described.

### 2.8. In Vitro Cytotoxicity Assay

Cytotoxic effect of blank nanoemulsion on several cell lines including NIH/3T3, 3T3-SA, RSC-96, RAW 264.7, RBL-2H3, CHO-K1, Caco-2, and NCI-H292 was determined using MTS assay according to the manufacturer’s protocol (Promega). Briefly, cells were trypsinized and plated into 96-well plate at density of 10^4^ cells/well. Cells were cultured overnight at 37 °C with 5% CO_2_. Cells were added with 20 mg/mL of blank nanoemulsion. Further, cells were incubated overnight and immediately added with 20 µL of MTS to each well. The cells were reincubated for 2 h at 37 °C with 5% CO_2_. The absorbance was measured at 490 nm using enzyme-linked immunosorbent assay (ELISA) microplate reader (Biorad, Hercules, CA, USA). The result was represented as percentage of cell viability, compared to untreated controls.

### 2.9. Acute Toxicity Study

Experimental procedures were carried out according to the World Health Organization (WHO) guidelines [14] and that of the Organization for Economic Co-operation and Development (OECD) for testing of chemicals, TG240 [15]. Five male and five female mice were given a single dose of 5000 mg/kg of the test substance. All mice were observed for toxicity signs, beharioral changes and mortality 30 min, 1, 2, 4, and 24 h after the administration. Similar observations were also performed once daily for 14 days. On day 15, all mice were fasted for 18 h and then sacrificed for gross anatomical examination of main internal organs. The organs were weighed, and observed macroscopically and microscopically. The microscopic observation was done through a standard histopatological examination using hematoxylin–eosin (HE) staining according to a standard protocol for HE staining on 5 μM of paraffin-embedded tissues [16,17].

### 2.10. Statistical Analysis

Statistical analysis was performed using one-way analysis of variance (ANOVA) for parametric data followed by post hoc method, and Mann–Whitney analysis for non-parametric data. A *p*-value < 0.05 was considered statistically significant.

## 3. Results and Discussion

Various processes take place during emulsification, including breakup of the droplets, adsorption of surfactant molecules, and droplets collision. These processes may occur simultaneously. Breaking of drops is feasible if the deforming force exceeds the Laplace pressure (*P_L_*), which is the interfacial force that acts against droplet deformation:

*P_L_* = γ (1/*R*_1_ + 1/*R*_2_). (1)

*R*_1_ and *R*_2_ are the smaller and the larger radii of the curvature of a deformed emulsion drop respectively, and γ is the interfacial force. Based on the Laplace equation [18], the droplet size is reduced with decreasing oil/surfactant ratio (increasing surfactant concentration). To form spontaneous nanoemulsion, not only is a high concentration of surfactant required, but so also is surfactant with a high HLB (hydrophilic and lipophilic balance) of over 12 [7]. Polyoxyl 40 hydrogenated castor oil used in this study shows a high HLB of between 14 and 16. Although non-ionic surfactant like PEG-40 hydrogenated castor oil is safer than ionic surfactant, applying a higher concentration in oral dosage form or via other routes of administration must be ensured, especially for a longer period of time, as reported here.

### 3.1. Physical Characteristics of Blank Nanoemulsion

**Figure 1:**
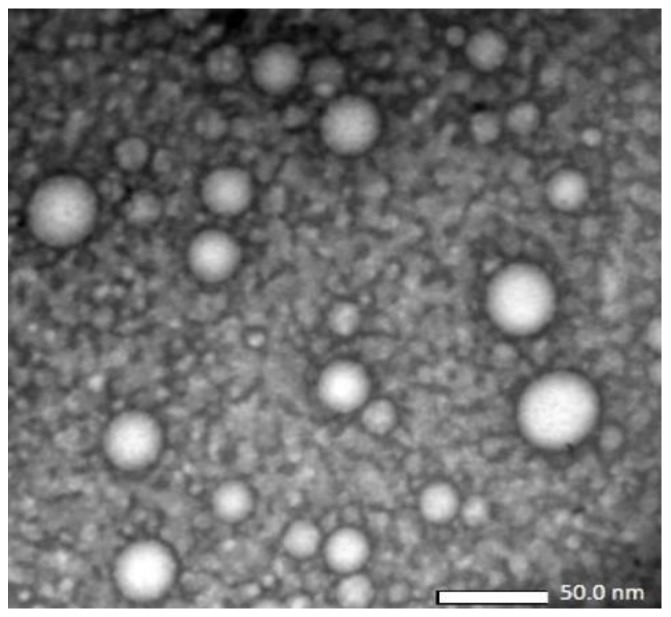
Cryo-transmission electron microscopic (cryo-TEM) observation of blank nanoemulsion (10,000× magnification).

The nanoemulsion produced a transparent dispersion due to smaller oil droplets, dispersed in the aqueous phase as confirmed by particle size measurement (Table 1) and microscopic observation (Figure 1).

**Figure 2:**
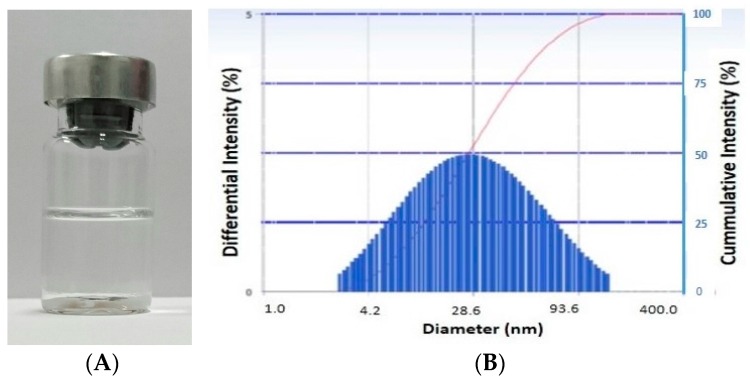
Visual appearance (**A**) and globule size distribution (**B**) of blank nanoemulsion after nine months storage at room temperature.

As clearly seen in Figure 1, the nanoemulsion showed a polydispersion system where most globules were at a size of <25 nm. The larger droplets of >25 nm are suggestive of smaller droplet coalescence. However, this phenomenon was controlled as confirmed by the transparent appearance of the nanoemulsion after a long storage period (nine months) at room temperature (Figure 2).

The negatively charged particle surface of the colloidal system (Table 1) is represented by the PEG-40 hydrogenated castor oil molecules covering the oil droplet surface. The negative charge was likely the result of the various hydrophilic groups of the non-ionic surfactant. The adsorption of non-ionic surfactant preferentially influences hydroxyl ions (OH^−^) on the surface of a droplet and therefore alters the zeta (ζ) potential of the droplet.

**Figure 3:**
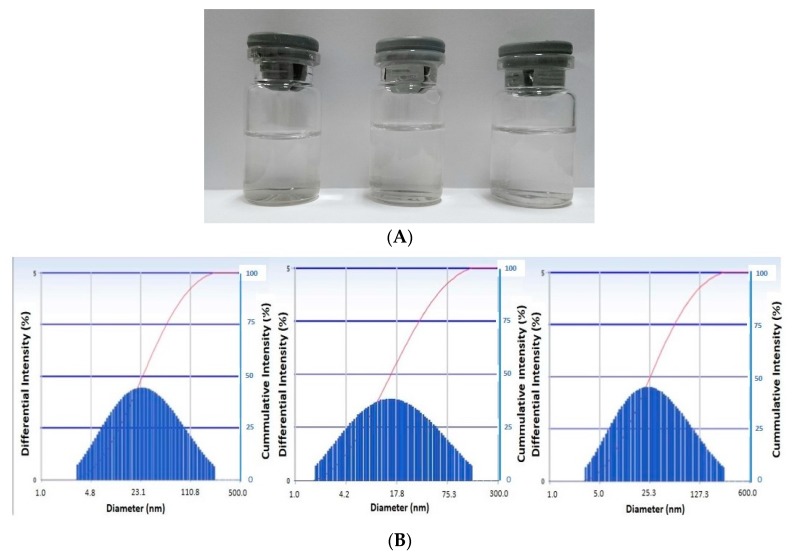
Visual appearance (**A**) and the globule size distribution (**B**) of blank nanoemulsion (left), blank nanoemulsion after being incubated with 0.1 N HCl 1:1 *v*/*v* (middle), and blank nanoemulsion after incubated with phosphate buffer (pH 6.8) 1:1 *v*/*v* (right). The incubation was done at 37 °C.

As presented in Figure 3, the nanoemulsion (a globule diameter of 20.9 nm, polydispersity index (PI, 0.394) is resistance to the GIT fluids. The nanoemulsion remained clear after incubation with either 0.1 N HCl (globule diameter of 19.6 nm, PI 0.084) or phosphate buffer pH 6.8 (globule diameter of 21.7 nm, PI 0.447) at 37 °C for 2 and 4 h, respectively. This indicated that no extensive coalescence occurred, which was confirmed by particle size analysis (PSA) data.

The stable dispersion system formed by this established formula was also shown (Figure 3) when the nanoemulsion was kept at room temperature for nine months. Likewise, no extensive coalescence was detected indicated by the transparency of the preparation, which was also confirmed by PSA data (globule diameter of 25.1 nm, PI 0.079).

### 3.2. Cytotoxicity Assay

**Figure 4:**
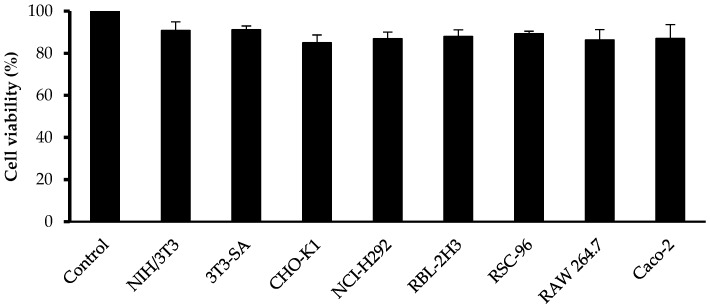
Cytotoxic effect on different cell lines.

Figure 4 presents cytotoxicity data in different cell lines. Blank nanoemulsion did not demonstrate any cytotoxic effect on NIH/3T3, 3T3-SA, RSC-96, RAW 264.7, RBL-2H3, CHO-K1, NCI-H292, and Caco-2 cell lines.

### 3.3. Acute Toxicity Analysis

The result of behavioral assessment is presented in Table 2. Following administration of as high as 5000 mg/kg of the test substance, neither abnormal behavioral changes nor death were observed during the entire 24 h obervation period. Thus, the lethal dose 50 (LD50) could not be observed at the dose used in the experiment.

There were no signs of straub, piloerection, ptosis, catalepsy, lacrimation, vocalization, salivation, tremor, convulsions, or writhing observed in the first 24 h after the administration of the test substance. This demonstrates that the test substance did not cause toxic effect on the central nervous system. Motor activity as well as body posture, respiration, urination, and defecation were normal. Furthermore, all mice showed normal reflexes, which indicates that the test substance did not affect spinal integrity in the central nervous system.

**Figure 5:**
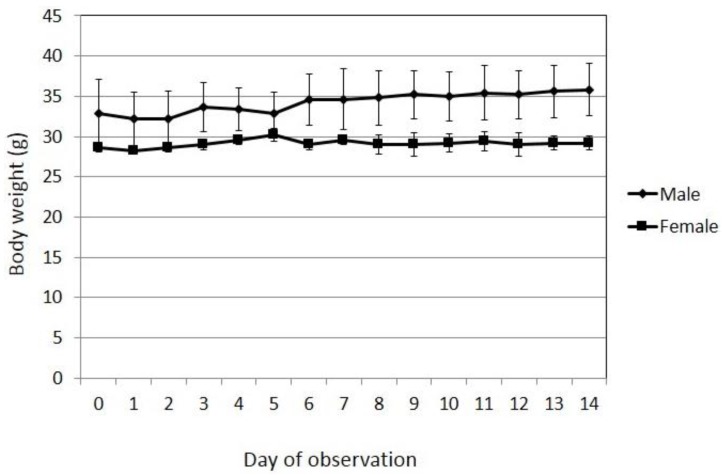
Fluctuation in the body weight of the test mice.

Figure 5 shows that, during the 14-day observation period following the administration of the test substance, the body weight of the test mice fluctuated within a normal range, and no marked decrease in body weight could be found. This finding shows that in general the test substance did not produce hazardous effects, and it had no effect on food intake. This is based on observation and is not significantly different when both genders are compared (*p* > 0.05).

**Figure 6:**
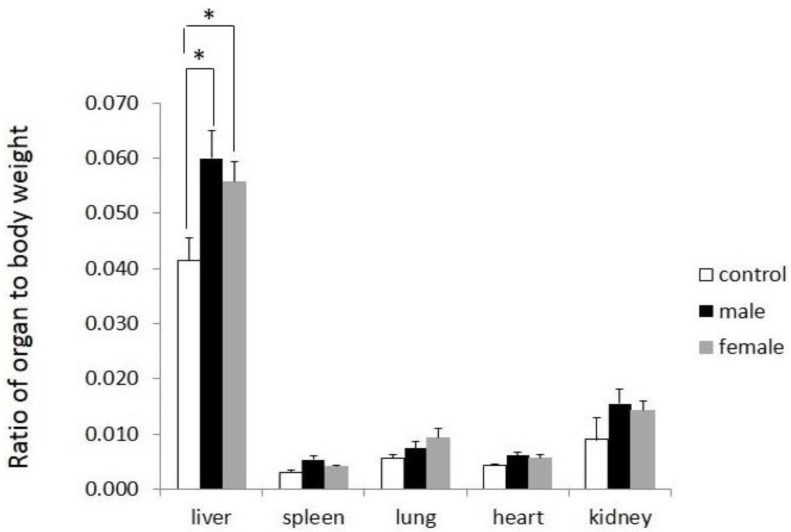
The organ-to-body weight ratios of the internal organs.

**Figure 7:**
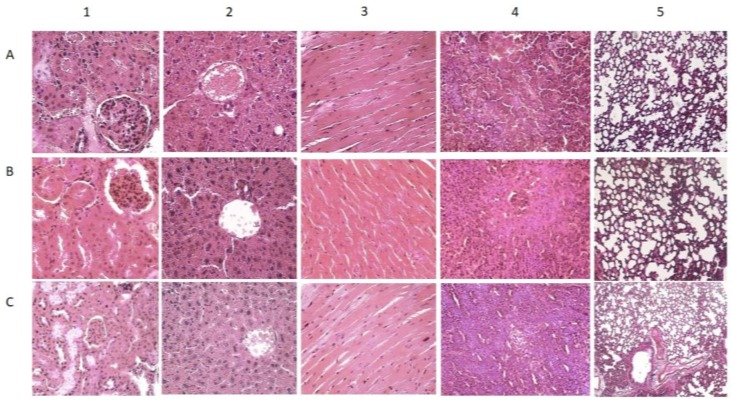
Histological results of the main organs—kidney (1), liver (2), heart (3), spleen (4), and lung (5)—from control (**A**), male (**B**) and female (**C**) mice receiving nanoemulsion. Magnification 100×.

As shown in Figure 6, the organ-to-body weight ratios of all internal organs examined were within a normal range. A minor significant difference was observed in the liver, but this was not followed by other studied parameters, including HE histological analysis. The histological examination was performed to evaluate organ damage upon treatment with a compound [19]. With regard to the histological profile of the main organs, as depicted in Figure 7, the results showed that there was no deviation from normal in all organs of test substance-treated mice (*p* > 0.5), which further corroborates the safety of the test substance.

## 4. Conclusions

Nanoemulsion composed with the component of an oil phase of GMO, PEG-40 hydrogenated castor oil as a surfactant, and PEG 400 as a co-surfactant (1:8:1) does not show any negative response when incubated in different types of cell lines. This in vitro data is confirmed in vivo after daily single-dose oral administration for 14 days. The use of high concentration of PEG-40 hydrogenated castor oil to form clear spontaneous and stable oil droplets when challenged in the GIT simulation fluids seems to be acceptable for the oral delivery of such active compounds loaded in the developed nanoemulsion.

## Figures and Tables

**Table 1:** Physical parameters of blank nanoemulsion.

Parameters	Value *
Particle size (nm)	21.4 ± 5.8
Polydispersity Index	0.245 ± 0.177
Zeta (ζ) potential (mV)	−10.6 ± 1.12

* Each parameter was determined in triplicates.

**Table 2:** Effects of the test substance on behavior following its single-high dose administration. A single dose of 5000 mg/kg was given to five mice of both sexes, followed by behavioral examination during a 24 h observation period.

Parameter			Observation Period (h)											
			Male						Female					
			0	0.5	1	2	4	24	0	0.5	1	2	4	24
Platform		*)	3.8	4.2	3.4	2.2	1.8	3.8	8.8	6.4	4.6	4.0	4.0	11.0
Motor Activity	Inrease	(%)	0	0	0	0	0	0	0	0	0	0	0	0
	Normal	(%)	100	100	100	100	100	100	100	100	100	100	100	100
	Descrease	(%)	0	0	0	0	0	0	0	0	0	0	0	0
	Immobile	(%)	0	0	0	0	0	0	0	0	0	0	0	0
Straub		(%)	0	0	0	0	0	0	0	0	0	0	0	0
Piloerection		(%)	0	0	0	0	0	0	0	0	0	0	0	0
Ptosis		(%)	0	0	0	0	0	0	0	0	0	0	0	0
Pineal reflex		(%)	100	100	100	100	100	100	100	100	100	100	100	100
Corneal reflex		(%)	100	100	100	100	100	100	100	100	100	100	100	100
Lacrimation		(%)	0	0	0	0	0	0	0	0	0	0	0	0
Catalepsy		(%)	0	0	0	0	0	0	0	0	0	0	0	0
Gait	Normal	(%)	100	100	100	100	100	100	100	100	100	100	100	100
	Abnormal	(%)	0	0	0	0	0	0	0	0	0	0	0	0
Front leg suspension		(%)	100	100	100	100	100	100	100	100	100	100	100	100
Establishment		(%)	100	100	100	100	80	100	100	100	100	100	100	100
Flexion		(%)	100	100	100	100	100	100	100	100	100	100	100	100
Hafner reflex		(%)	100	100	100	100	100	100	100	100	100	100	100	100
Mortality		(%)	0	0	0	0	0	0	0	0	0	0	0	0
Grooming		(%)	20	20	0	0	20	60	20	60	40	40	0	40
Defecation		(%)	40	20	40	40	60	20	100	80	60	20	60	100
Urination		(%)	40	20	40	40	60	40	60	60	20	20	20	40
Respiration	Rapid	(%)	0	0	0	0	0	0	0	0	0	0	0	0
	Normal	(%)	100	100	100	100	100	100	100	100	100	100	100	100
	Short	(%)	0	0	0	0	0	0	0	0	0	0	0	0
Salivation		(%)	0	0	0	0	0	0	0	0	0	0	0	0
Vocalization		(%)	0	0	0	0	0	0	0	0	0	0	0	0
Tremor		(%)	0	0	0	0	0	0	0	0	0	0	0	0
Convultion		(%)	0	0	0	0	0	0	0	0	0	0	0	0
Writhing		(%)	0	0	0	0	0	0	0	0	0	0	0	0

*) The average number of head dipping; % = Percent of number of animals.
